# An Integrated Low-Power Lock-In Amplifier and Its Application to Gas Detection

**DOI:** 10.3390/s140915880

**Published:** 2014-08-27

**Authors:** Paulina M. Maya-Hernández, Luis C. Álvarez-Simón, María Teresa Sanz-Pascual, Belén Calvo-López

**Affiliations:** 1 Electronics Department, Instituto Nacional de Astrofísica, Óptica y Electrónica (INAOE), Luis Enrique Erro #1, Tonantzintla 72840, Puebla, Mexico; E-Mails: paulina_maya@inaoep.mx (P.M.M.-H.); lucas@inaoep.mx (L.C. Á.-S.); 2 Group of Electronic Design, Aragon Institute for Engineering Research, I3A, Facultad de Ciencias, Pedro Cerbuna 12, Zaragoza 50009, Spain; E-Mail: becalvo@unizar.es

**Keywords:** analog sensor conditioning, lock-in amplifier, phase-sensitive detection, gas sensing, portable applications

## Abstract

This paper presents a new micropower analog lock-in amplifier (LIA) suitable for battery-operated applications thanks to its reduced size and power consumption as well as its operation with single-supply voltage. The proposed LIA was designed in a 0.18 μm CMOS process with a single supply voltage of 1.8 V. Experimental results show a variable DC gain ranging from 24.7 to 42 dB, power consumption of 417 μW and integration area of 0.013 mm^2^. The LIA performance was demonstrated by measuring carbon monoxide concentrations as low as 1 ppm in dry N_2_. The experimental results show that the response to CO of the sensing system can be considerably improved by means of the proposed LIA.

## 1. Introduction

The sensor market is undergoing continuous development to satisfy the increasing demand for portable sensing applications, mainly for healthcare, environmental and industrial monitoring [1–4]. In fact, portability significantly widens the spectrum and scenarios of sensing applications, but requires optimization of the sensor system at all levels, from transducer developments to the design of power-efficient electronic interfaces. Only in this way is it possible to attain a system showing the required low-voltage single-supply operation for compatibility with low-form batteries, low power consumption to extend the life of the battery and small size to ensure portability. In the case of remote sensing systems, such as wireless sensor networks (WSNs), the deployment impact must also be minimized.

Unfortunately, the small signal provided by low voltage low power sensors may be obscured by noise and interference signals, thus requiring special amplification techniques to increase the signal-to-noise ratio [5]. In particular, lock-in amplifiers (LIAs), based on a technique known as phase sensitive detection, are the most common choice to recover weak signals from noise by singling out the component of the signal at a specific reference frequency and phase [6]. Although widely used in instrumentation, the limitations of commercial LIAs in terms of size, cost and power consumption prevent their use within portable measurement systems. Moreover, LIAs are not currently marketed in an integrated manner most likely because, if integrated with the sensor, the product would turn into a specific System-On-Chip instead of a general purpose sensor, increasing the cost and reducing profit margins. However, preliminary results towards the design of an integrated LIA both found in literature and presented in this paper are quite promising and open up a really interesting research field. In the literature, only a few integrated versions can be encountered [7–14]: some of them operate in dual supply mode [7,8], whereas those with single supply exhibit power consumption in the mW range [9–14], jeopardizing its use in battery operated systems. It is noteworthy that all of these implementations are based on a voltage mode approach using rather complex building blocks and do not specifically focus on low-voltage low-power operation to meet the requirements of portable systems. Besides, they are designed to match specific applications, which limits their use to a given operation frequency and prevents them from being used in general purpose conditioning circuits.

Taking all this into consideration, the goal of the paper is the design of a versatile analog current-mode LIA fulfilling the requirements of portable systems, namely low voltage single supply and reduced area and power consumption, while preserving good recovery capability. The proposed topology was first introduced by the authors at a conference [15], where preliminary results were shown. This work comprises a detailed explanation of the circuit design and operation as well as an exhaustive experimental characterization of the circuit. The current mode lock-in amplifier, integrated in a 1.8 V–0.18 μm standard CMOS process, arises as a low cost solution, compatible with mixed analog-digital signal systems. To show its functionality, the proposed LIA is used to detect low concentrations of carbon monoxide (CO) with a commercial chemiresistive sensor based on tin dioxide [16]. This kind of sensor was selected because chemiresistive metal-oxide (MOX) structures are compatible with integrated CMOS microsystem technologies, since they can be co-integrated with an appropriate intra-CMOS process, facilitating the forthcoming accomplishment of fully integrated low-cost portable smart gas sensing devices [17–20].

The paper is organized as follows. Section 2 presents the proposed lock-in amplifier and describes its basic building blocks. Section 3 reports the main experimental results. Section 4 demonstrates the circuit capability to improve signal-to-noise ratio by measuring concentrations of carbon monoxide down to 1ppm. Finally, some conclusions are drawn in Section 5.

## 2. Lock-In Amplifier Architecture

**Figure 1.**
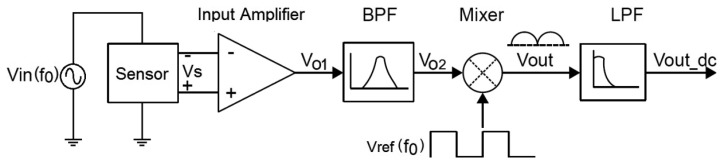
Block diagram of a typical lock-in amplifier.

The block diagram of a sensor readout system using a typical analog lock-in amplifier is shown in Figure 1. A sensor is excited by a sinusoidal signal with a known frequency *f*_0_ and its response *V_s_* is injected into the lock-in system. The first active block is a low noise amplifier which provides high gain. It may be followed by a band-pass filter to remove or attenuate the noise contribution at all the frequencies except for *f*_0_. The next block is a mixer or Phase-Sensitive Detector (PSD), which multiplies the modulated input signal by a square reference signal *V_ref_*. By properly synchronizing the sensor and control signals to have the same frequency and phase values, the data signal is full-wave rectified at the output. In this way, the DC component may be easily extracted by means of a low-pass filter (LPF) with a suitable low cut-off frequency, and is given by *V_out_dc_* = 2 *V_s_V_ref_*.cos*θ*/*π*, where *θ* is the phase difference between *V_s_* and *V_ref_*.

**Figure 2.**
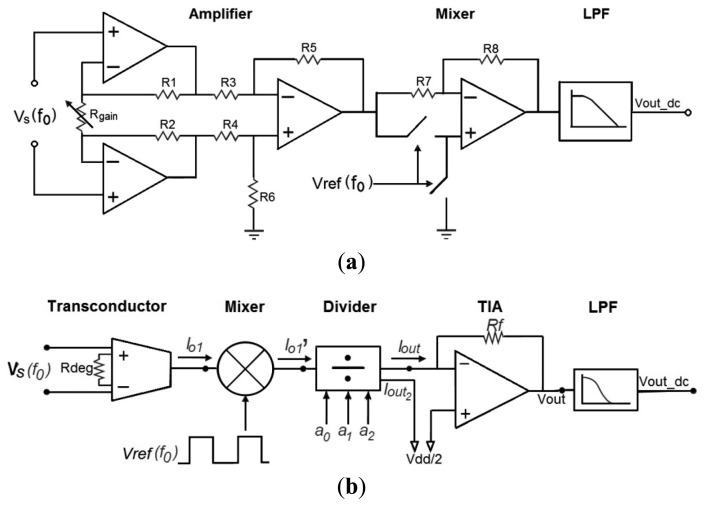
Lock-in amplifier schemes: (**a**) conventional voltage-mode approach and (**b**) proposed topology.

In most integrated LIAs found in literature, the input amplifier is a classic 3-opamp instrumentation amplifier whose gain is controlled through an external resistor *R_gain_*, while the mixer is usually based on an dual supply opamp voltage amplifier stage with a switch-controlled ±1 gain, as shown in Figure 2a. The LIA proposed in this paper totally differs from this approach. In order to accomplish a small and power saving solution compatible with low bias voltage, a current mode approach is adopted [21–24]. Hence, the rectification of the signal is carried out in the current domain and gain adjustment is achieved through a 3-bit digitally programmable current divider. As shown in Figure 2b, our proposal consists of a voltage-to-current converter that translates the voltage signal into a current signal. It is followed by a resistorless current mixer, which performs the phase-sensitive detection. A MOS current divider provides gain programmability to the system. Finally, a transimpedance amplifier converts the signal back into a voltage mode for further processing. By using this architecture, not only is the number of active blocks reduced but also the number of resistors, which consume otherwise most of the area and add noise to the circuit. Note that the proposed LIA must be powered by a single supply, which implies that the common mode is related to *V_dd_*/2. Each building block of the proposed LIA is briefly described below at transistor level.

### 2.1. Transconductor

**Figure 3.**
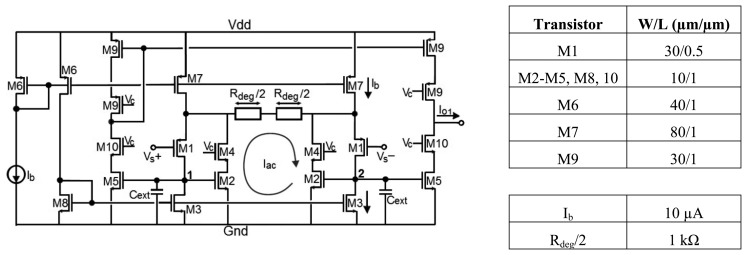
Input stage: g*_m_*-boosting source degenerated transconductor.

The input stage is a voltage to current converter to handle the signal of interest in the current domain. The chosen transconductor is a differential pair with source degeneration, which is one of the most popular transconductors. The requirement that the input pair transconductance *g_m_* is much higher than the inverse of the degeneration resistance *R_deg_* to attain a linear transfer characteristic inversely proportional to *R_deg_* is usually satisfied by employing large transistors and/or bias currents. However, this is not desirable in terms of area and power consumption. As a solution to improve the power efficiency while obtaining high linearity, a simple technique has been adopted based on the boost of the input pair transconductance by means of negative feedback [25], as shown in Figure 3.

Input transistors M1 act as improved voltage followers, buffering the differential input voltage $V_{s}=V_{s}^{+}-V_{s}^{-}$ to the terminals of the degeneration resistor *R_deg_*. The linearized differential current signal *I_ac_* = *Vs*/*R_deg_* can be easily copied out by loading nodes 1 and 2 with transistors M5. Active cascodes (M2-M4, M5-M10 and M9) are used in order to maximize the output impedance and enhance the *g_m_*-boosting action, thus increasing the accuracy of the total transconductance *G_m_*=1/*R_deg_* of the system. Two linear poly-silicon resistors *R_deg_*/2 =1 kΩ were used to give more symmetry to the layout of the circuit. Finally, through a high swing cascode current mirror (M9), the differential current is converted into a single-output current given by *I_o_*_1_=2*V_s_*/*R_deg_*.

For this stage, note that the dominant pole is located at nodes 1 and 2, so external capacitors *C_ext_* can be added as shown in Figure 3 to adjust the input stage bandwidth and thus reduce the noise contribution.

### 2.2. Mixer

The mixer function is to carry out the phase sensitive detection over the signal *I_o_*_1_ according to the reference signal *V_ref_*. For this purpose, a current mode circuit where *V_ref_* determines the direction of the current flow is used to provide a fully rectified output current with a DC component proportional to the signal of interest and (almost) independent of noise.

**Figure 4.**
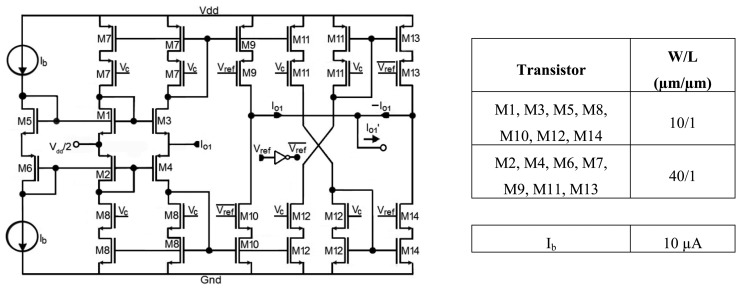
Resistorless current mixer.

The topology employed, as depicted in Figure 4 [26], operates as follows: when the square reference signal *V_ref_* is low (*V_ref_* = 0, $\bar{V_{\text{ref}}}=1$), the input current *I_o_*_1_ is copied through the high swing cascode current mirrors M7-M9 and M8-M10 so that $I_{o1}^{\prime }=I_{o1}$, acting as a current follower; when *V_ref_* is high (*V_ref_* = 1, $\bar{V_{\text{ref}}}=0$), the input current is copied and inverted through the paths M7-M11-M12-M14 and M8-M12-M11-M13, so that $I_{o1}^{\prime }=-I_{o1}$, this time acting as a current inverter. With the reference signal in phase with the input signal, the result is a full rectification of the input current signal, and the operation of the mixer can be represented by $I_{o1}^{\prime }={\left(-1\right)}^{V\text{ref}}I_{o1}$.

### 2.3. Current Divider

**Figure 5.**
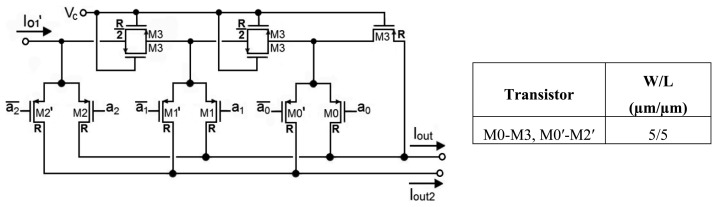
3-bit MOS current divider.

Gain programmability is achieved through a 3-bit MOSFET R-2R current divider (M-2M divider) as shown in Figure 5 [27], where each PMOS transistor is equivalent to a resistance R.

The rectified current $I_{o1}^{\prime }$ is driven to the M-2M ladder, where it is divided into two output currents, $I_{\text{out}}=\Delta I_{o1}^{\prime }$ and $I_{\text{out}2}=(1-\Delta )I_{o1}^{\prime }$. The division factor Δ, controlled by a 3-bit digital word *A*(3)={*a*_2_, *a*_1_, *a*_0_}, is given by:

(1) $$\Delta =\frac{1}{2^{n}}\left[1+\sum_{j=0}^{n-1}\left(2^{j}-a_{j}2^{j}\right)\right]\text{with}\;n=3$$

The output current *I_out_* is connected to the virtual ground (*V_dd_*/2) input of the next block, the transimpedance amplifier, while the complementary output, *I_out_*_2_, is grounded to *V_dd_*/2 for proper current division. The advantage of using parallel transistors to generate R/2 and one transistor to generate R is that the voltage drop between the input node and the current output nodes is smaller than it would be with two transistors in series to generate 2R and one transistor to generate R [28].

### 2.4. Transimpedance Amplifier

**Figure 6.**
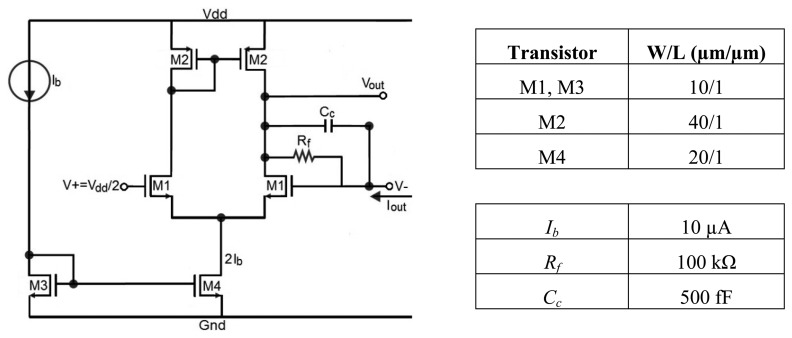
Transimpedance amplifier.

The signal of interest is converted back to the voltage domain by using a transimpedance amplifier (TIA). The topology is shown in Figure 6. It consists of a single-stage differential amplifier and a linear poly-silicon feedback resistor *R_f_* = 100 kΩ value and a compensation capacitor *C_c_* = 500 fF. If *I_out_* is the current delivered by the current ladder, the output voltage is given by *V_out_* =−*I_out_R_f_*.

Considering the cascade connection of the blocks presented so far, the total gain of the system as a function of the 3-bit digital word is given by the following expression:

(2) $$\frac{V_{\text{out}}}{V_{s}}=-\frac{R_{f}}{R_{\text{deg}}}\left(\frac{1}{2^{n}}\left[1+\sum_{j=0}^{n-1}\left(2^{j}-a_{j}2^{j}\right)\right]\right){\;(-1)}^{V_{\text{ref}}}\text{with}\;n=3$$

That is, for a given *V_ref_* the system is equivalent to a programmable gain amplifier whose gain depends on the *R_f_* to *R_deg_* ratio and on the current ladder digital word. Note that both *R_f_* to *R_deg_* are implemented with the same material, a high resistivity poly-silicon (HRP) layer, so both will suffer the same variations with process and temperature, thus ensuring good accuracy and robustness. Furthermore, current division is highly linear [27], and so is the whole system.

### 2.5. Low Pass Filter

The low-pass filter (LPF) is an RC passive implementation with 5 Hz cut-off frequency. Since this circuit is responsible for removing the harmonic components of the noise and interference signals, its cutoff frequency should be as low as possible. As usually found in literature, the LPF was externally connected to the circuit.

### 2.6. Simulation Results

**Figure 7.**
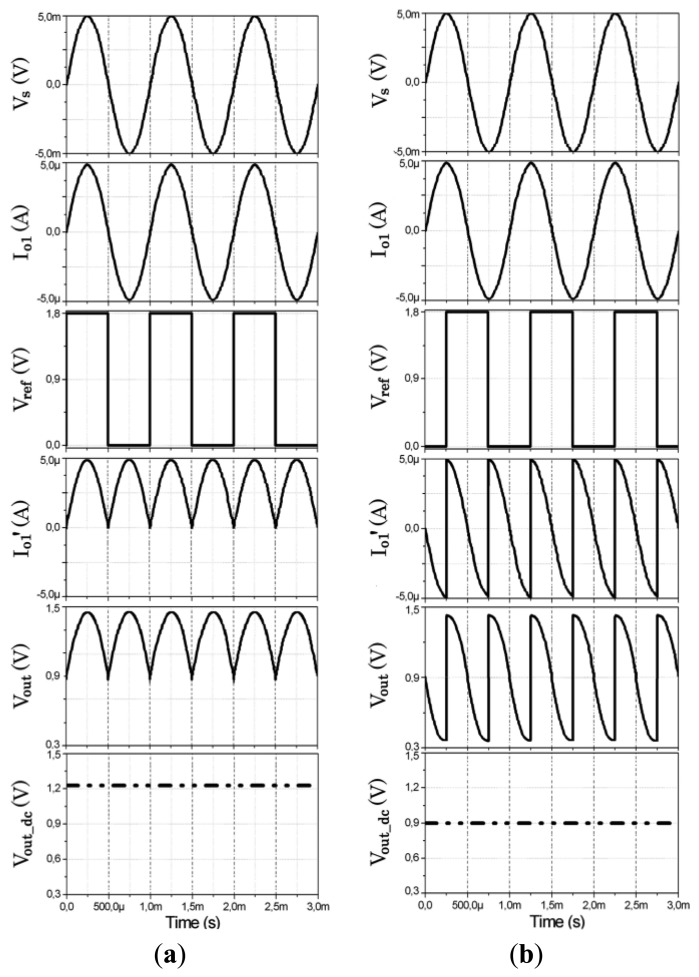
Performance of LIA: (**a**) *V_s_* and *V_ref_* in phase; (**b**) *V_s_* and *V_ref_* with a phase shift of 90°.

Some post-layout simulation results are shown in Figure 7 to illustrate the basic operation principle of the proposed LIA. A sinusoidal signal *V_s_* is introduced to the input of the transconductor and converted into the *I_o_*_1_ output. The current mode mixer multiplies the signal *I_o_*_1_ and the reference square signal *V_ref_*. If *I_o_*_1_ and *V_ref_* are in phase, as shown in Figure 7a, the result is a fully rectified current signal $I_{o1}^{\prime }$, which is converted into a voltage signal *V_out_*, whose DC component is extracted by the LPF. As the input signal is fully rectified, the DC component *V_out_dc_* is proportional to its amplitude. In contrast, if *I_o_*_1_ and *V_ref_* are 90° out of phase, as shown in Figure 7b, the result is a voltage signal *V_out_* whose DC component is equal to the common-mode voltage *V_dd_*/2, independently of the input signal amplitude. Therefore, no information can be extracted in this case.

## 3. Lock-In Amplifier Experimental Performance

**Figure 8.**
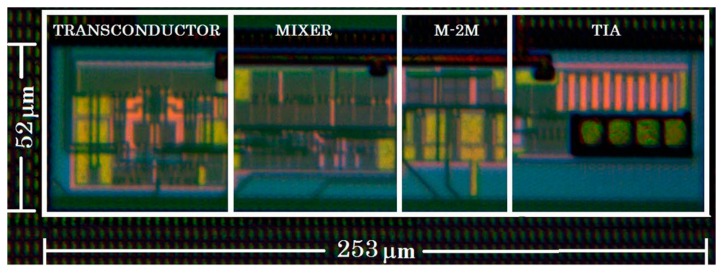
Photograph of the fabricated lock-in amplifier.

The proposed LIA was designed and integrated in standard 0.18 μm CMOS technology with 1.8 V supply voltage. The photograph of the integrated circuit is shown in Figure 8. The required area is only 0.013 mm^2^ which, together with the low power consumption, 417 μW, makes the circuit suitable for portable applications.

The gain programmability provided by the 3-bit M-2M ladder is reported in Table 1, which shows the ideal gain (*A_id_*) according to Equation (2) and the experimentally measured gain (*A_exp_*).

**Figure 9.**
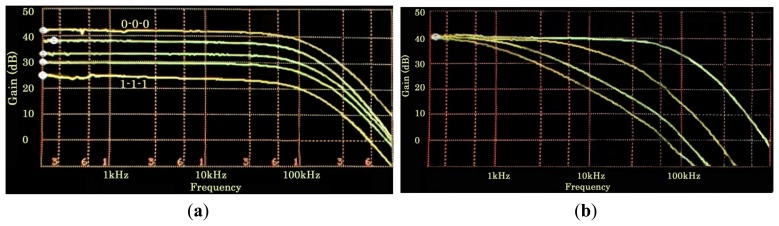
(**a**) Gain programmability and (**b**) BW variation with *C_ext_*.

Figure 9a shows the frequency response of the programmable LIA at different gain settings (*A* = {000,010,100,101,111}). As previously mentioned, the bandwidth of the system can be adjusted by means of *C_ext_* in order to limit the noise. This feature can be seen in Figure 9b, which shows the transfer function at maximum gain for different *C_ext_* values: 7.5 pF, 470 pF, 2.2 nF and 4.7 nF to set a bandwidth of 100 kHz, 6 kHz, 1.3 kHz and 0.6 kHz, respectively. Since no on-chip calibration circuit was included in the design, experimental bandwidths were smaller than the simulated ones due to parasitic capacitances, mainly from chip packaging. In brief, programmability can be used to adjust the gain in accordance with the input amplitude, while bandwidth can be adjusted according to the input frequency through the adequate selection of *C_ext_*.

**Figure 10.**
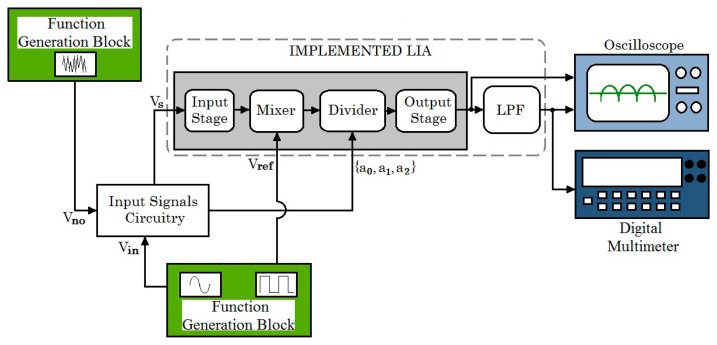
Experimental setup block diagram.

Figure 10 shows the experimental setup block diagram utilized to evaluate the dynamic performance of the integrated LIA. The input (*V_in_*), reference (*V_ref_*) and noise (*V_no_*) signals were generated by a Function Generator, so the phase between the noisy input signal *V_s_* = *V_in_*+ *V_no_* and the reference signal *V_ref_* was externally regulated. The rectified signal *V_out_* was monitored with an oscilloscope and the DC output level *V_out_dc_* after the low-pass filter with (cut-off frequency of 5.16 Hz) was measured with a 6½ digit digital multimeter.

**Figure 11.**
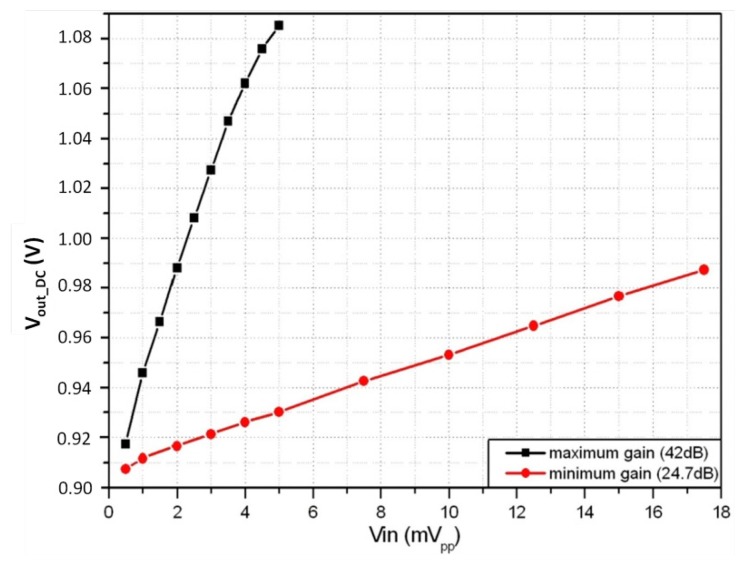
Output DC voltage *vs.* input signal at maximum and minimum gain.

First, proper operation of the LIA was checked by measuring the output when processing a 1 kHz noise-free sinusoidal signal. It was found that the full-scale input and output ranges are 5 mV_pp_ and 630 mV_pp_, respectively, with a resolution of 50 μV_pp_ and a sensitivity of 40 mV/mV_pp_ for maximum gain; for the minimum gain the full-scale input and output ranges are 17.5 mV_pp_ and 300 m*V_pp_* with a resolution of 120 μV_pp_ and a sensitivity of 8 mV/mV_pp_. Figure 11 shows the measured output DC voltage *vs.* the peak-to-peak input voltage for the maximum and minimum LIA gain.

**Figure 12.**
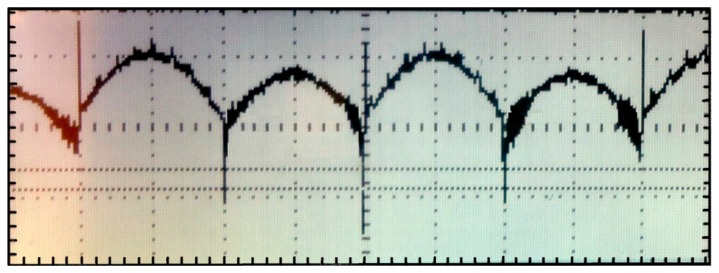
Rectified output (*x*-axis: 250 μs/div, *y*-axis: 500 mV/div).

Figure 12 shows, for a noise-free sinusoidal input signal of 5 mV_pp_ at 1 kHz in phase with the square reference signal, and maximum amplifier gain (*A*_exp_ = 42 dB), an oscilloscope screenshot of the rectified signal *V_out_* =346.2 mV_p_. The voltage after the LPF was measured with the multimeter, obtaining *V_out_dc_* =1.0852 V *vs.* the ideal *V_out_dc_id_* =1.1003 V, which means a 1.9% relative error for the maximum input voltage that can be handled at maximum gain. This relative error remains lower than 0.8% for input voltages equal to or lower than 4.5 mV_pp_.

Next, the capability of the circuit to recover information from signals buried in noise was evaluated. Two noise cases were studied: a single-frequency interference and white noise. For the first case the signal *V_s_* was a sinusoidal voltage of 50 μV_pp_ at 1 kHz frequency with an interfering sinusoidal signal at different frequencies ranging 1.002 to 2 kHz and an amplitude *V_no_* = 3.2 mV. For the second case the signal *V_s_* was a sinusoidal voltage of 50 μV_pp_ at 1 kHz frequency buried in Gaussian white noise, characterized by a root mean square value *V_no_rms_* = 2.3 mV. The noise bandwidth was set to 10 MHz, so that it fully covers the signal frequency range used in this study. The noise levels were provided by the Function Generator and added to the input signal by means of a wide bandwidth operational amplifier in an adder configuration.

The output voltage for a noise-free signal of *V_s_* =50 μV_pp_, *V_out_dc_exp_*, and the output *V_out_dc_noisy_* for a noisy signal are reported in Table 2 for both types of noise and two different *C_ext_*, that is, two different bandwidths. The relative error when recovering signal amplitudes for SNR levels down to −42.13 dB is below 5.0% with *BW_LIA_* = 125 kHz and below 4.1% with *BW_LIA_* = 13 kHz. Measurements confirm the capability of the proposed topology to recover signals highly buried in noise.

As can be seen from Case 1 in Table 2, the relative error in the measurement increases when the frequency of the interfering signal gets closer to the frequency of *V_s_*; the dead band, *i.e.*, the frequency range in which the recovery error increases significantly (over 2.9%), is about 2 Hz. When the frequency of the interfering signals is very close to the reference signal frequency, the undesired signals manage to pass the acceptance band of the output filter and are detected by the LIA with a phase difference, thus contributing to the DC voltage. For Case 2, since white noise contains signals within all the frequency range, some signals will match the frequency of the reference signal but with a phase difference and, again, get to contribute to the output DC voltage. The relative error can be reduced down to 4.1% by reducing the bandwidth to 13 kHz with the external capacitors.

**Figure 13.**
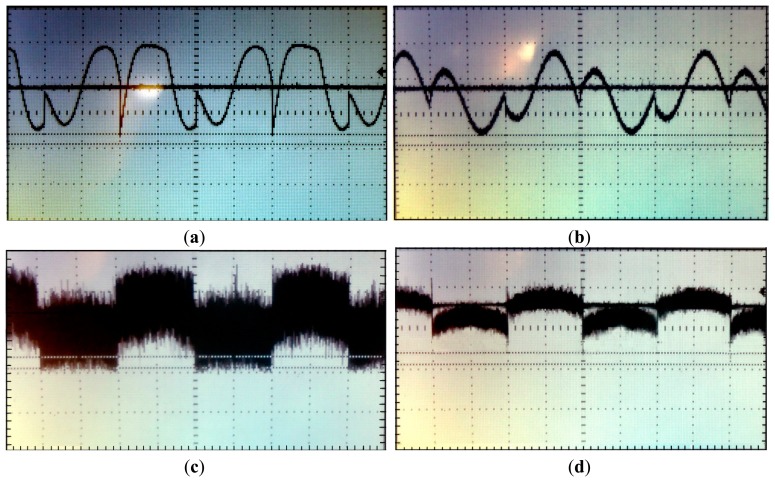
Rectified output and DC extraction (*x*-axis: 250 μs/div, *y*-axis: 500 mV/div): (**a**) *V_s_* signal with sinusoidal interfering signal, Case 1a with *BW_LIA_* = 125 kHz; (**b**) *V_s_* signal with sinusoidal interfering signal, Case 1a with *BW_LIA_* = 13 kHz; (**c**) *V_s_* signal with Gaussian white noise, Case 2 with *BW_LIA_* = 125 kHz; (**d**) *V_s_* signal with Gaussian white noise, Case 2 with *BW_LIA_* = 13 kHz.

Figure 13 displays some examples of the LIA waveforms for some of the noise tests. Figure 13a,b shows the rectified output and DC output for the interfering signal of Case 1a, with *BW_LIA_* = 125 kHz and *BW_LIA_* = 13 kHz, respectively. Figure 13c,d shows the rectified output and DC output obtained by measuring the amplitude of the input signal submerged in white noise of Case 2 with *BW_LIA_* = 125 kHz and *BW_LIA_* = 13 kHz, respectively. In the first case, despite the bandwidth reduction of the LIA, because the interference signal is operating very close to the signal of interest it is not possible to filter it so as to attenuate it significantly. In the second case, even when the signal of interest is immersed in white noise, the LIA is able to detect it, being the attenuation of the noise through filtering more evident, lessening the error from 5% to 4.1%. In both cases, even when the signal of interest is deeply submerged in noise, the LIA manages to extract the DC contribution of the signal of interest with a small margin of error, besides being able to reduce the relative error of the measured data by increasing the value of the external capacitors.

Finally, in Table 3 the main characteristics of the LIA are summarized and compared with recent integrated implementations found in the literature. It can be noticed that the proposed topology, operating under single supply voltage, exhibits a significant reduction in power consumption and area of integration. Since our proposal does not include phase control, to perform a fairer comparison note that a dual-phase LIA configuration to eliminate phase dependency would have an estimated power consumption of about 0.84 mW and area around 0.026 mm^2^, still resulting in a very competitive solution when compared to previous implementations. It is worth noting that the circuit is not subject to a specific operating frequency, so it can be used to operate at the frequency that the user requires as long as it lies within the bandwidth of the amplifier. In summary, compared to its voltage-mode counterparts, the proposed current-mode LIA shows wider bandwidth, good capability to recover signals submerged in noise (SNR = −42.13 dB) and a reduction of one to two orders of magnitude in power consumption and integration area, which validates the design strategy.

## 4. Detection of Small Gas Concentrations

### 4.1. Gas Sensor

**Figure 14.**
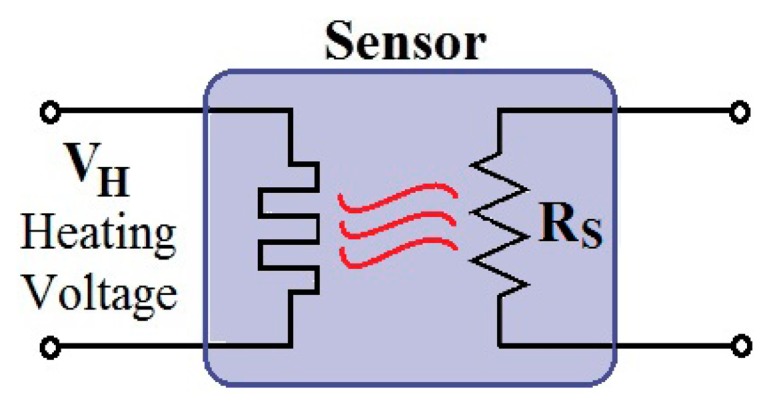
Circuit representation of a metal oxide semiconductor gas sensor.

Metal oxide semiconductor sensors are extensively used in gas detection applications [29,30]. The sensing layer, a metal oxide-based thick film on a Si-micromachined substrate, is sensitive to a specific set of gases when heated at high temperature, usually 200–400 °C. Its electrical resistance changes as a consequence of gas adsorption, being the sensitivity and selectivity determined by the operating temperature. Therefore, a micro-heater is needed to set a constant and uniform temperature and thus ensure good performance of the sensor [31]. The circuit representation of a metal oxide semiconductor gas sensor is shown in Figure 14.

To show the functionality of the proposed LIA, a CO gas detection system was implemented in the laboratory. The commercial Carbon Monoxide Sensor AS-MLC, based on a tin dioxide sensitive layer, was used [16]. Heater and interdigital electrode structures are positioned on a 1 μm-thin membrane on top of which the tin dioxide is deposited, thus creating a gas concentration dependent resistance (R_S_). Some of the benefits of this sensor are its high sensitivity to CO (0.5 to 500 ppm), low power consumption, long lifetime, low cross-sensitivity and long-term stability.

The typical operation temperature of the sensor is 270 °C with approximately 2.2 V heater voltage and 35 mW power consumption. However, in a practical implementation, pulse width modulation (PWM) can be applied to the heater to significantly reduce energy consumption [32]. The conditioning circuit, in turn, should consume negligible power when compared to the heater in order not to contribute to the power consumption of the whole system and thus extend its life time.

### 4.2. Experimental Characterization

**Figure 15.**
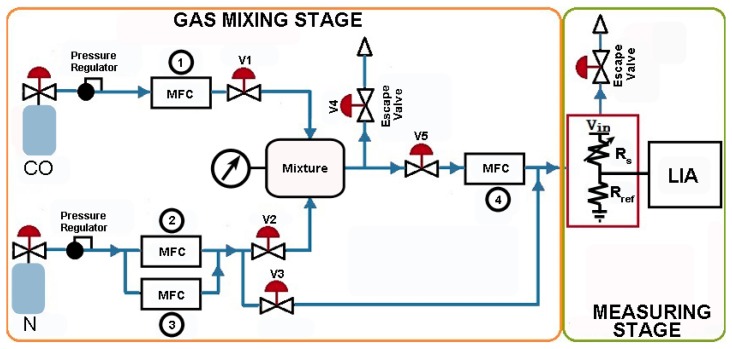
Experimental setup scheme.

The proposed lock-in system was tested within an experimental setup to detect small concentrations of carbon monoxide with the above described AS-MLC gas sensor. The setup, shown in Figure 15, ensures well defined gas concentration levels through the Mass Flow Controllers (MFCs). A mixture of CO plus N_2_ is prepared at the mixing stage and the CO concentration in the gas chamber is measured without and with the proposed LIA. The detection of CO was carried out in a controlled N_2_ environment to detect only the changes in sensor resistance due to the small variations of CO and avoid cross-sensitivity due to the presence of other gases.

**Figure 16.**
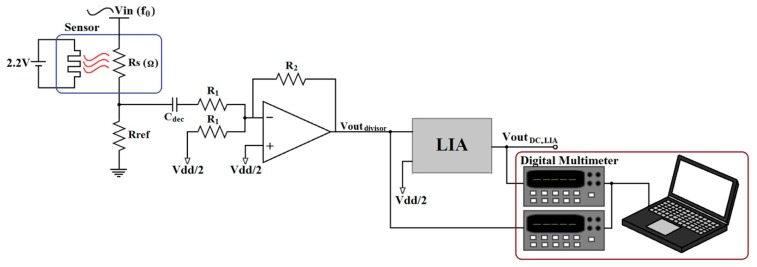
Measurement system with lock-in amplifier.

**Figure 17.**
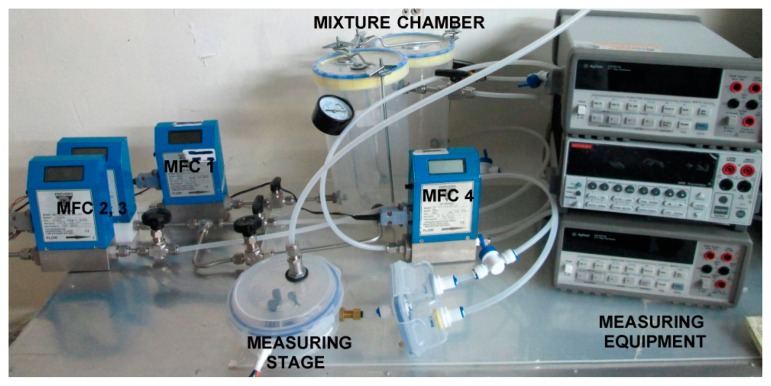
Physical implementation of the experimental setup.

The measurement system is shown in Figure 16. The sensor resistance *R_s_* is placed into a voltage divider stimulated with a sinusoidal voltage *V_in_* =15 mV_pp_ at 1 kHz frequency and with a reference resistor of 51kΩ. A decoupling capacitor *C_dec_* followed by an analog adder was used to set the required DC level (V_dd_/2) at the LIA's input. The LIA was set to its minimum gain (24.7 dB) and BW = 13 kHz. The physical implementation of the experiment can be seen in Figure 17.

**Figure 18.**
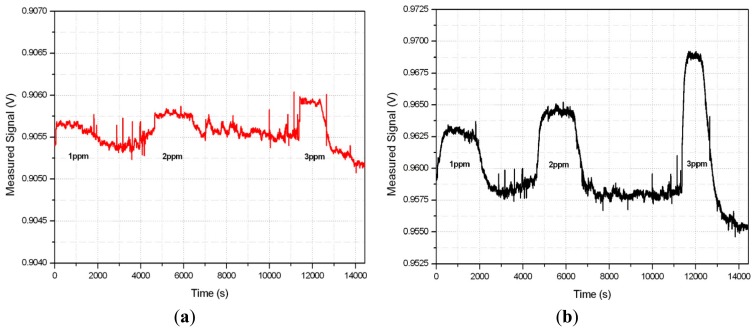
Output Voltage for different CO concentrations: (**a**) Voltage divider; (**b**) Lock-in amplifier.

Three different concentrations of carbon monoxide with nitrogen (CO + N_2_), namely 1, 2 and 3 ppm, were introduced to the sensor measurement chamber, alternating it with a nitrogen flux to provide some recovery time to the sensor. Although the sensor would usually be operated in atmospheres containing oxygen, experimental tests with nitrogen provide information about the intrinsic noise of the sensor and validate the functionality of the lock-in amplifier. The temperature in the chamber was 22.7 °C and relative humidity was 12.3%. Both the temperature and the humidity were monitored by a temperature and humidity sensor [33] during the measurements because they have an impact on sensor sensitivity but, as this is a commercial sensor, it is out of the scope of this work to characterize that dependence. However, it should be noted that the presence of oxygen and humidity in the measurement process are a source of noise, so that for future measurements they should be taken into account. In Figure 18, the output signal of the voltage divider (Figure 18a) and at the output of the LIA (Figure 18b) for different concentrations of gas is shown. The sensitivity of the system without LIA was 0.15–0.18 mV/ppm while the sensitivity when the LIA was used increased to 3–4.5 mV/ppm. It is observed from Figure 18a that when no LIA is used it is difficult to distinguish whether CO is present for low concentrations, as noise levels are in the same order as the signal to be measured. In contrast, the use of the lock-in amplifier makes it possible to directly discriminate and determine the presence of low gas concentrations. Although this kind of microsensor is used mainly for specific applications, such as safety, where small concentrations of CO, which is highly toxic, must be detected, the system we set up could detect up to 40 ppm. This measurement process was completed with two different LIA prototypes showing similar results, which validates the reliability of the proposed solution.

**Figure 19.**
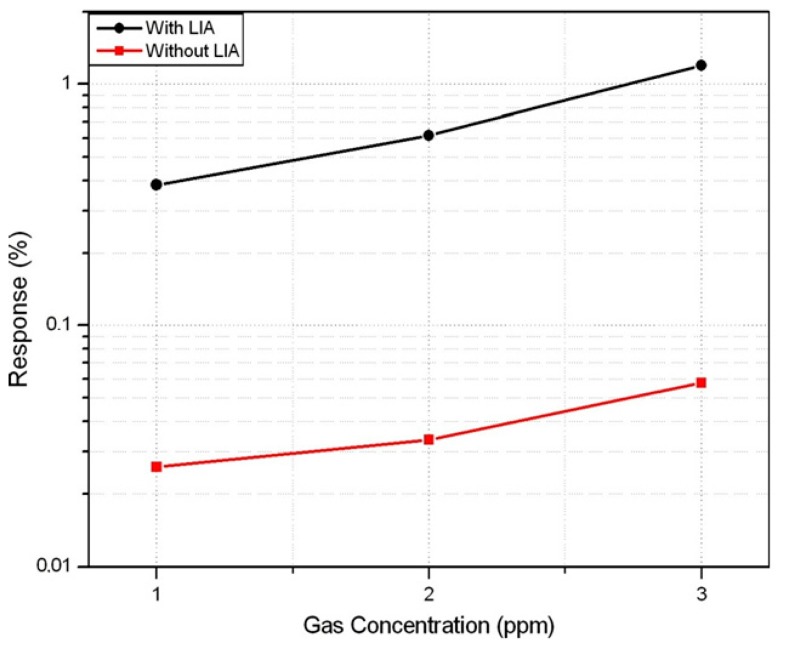
Response to CO with and without LIA.

The response in % to CO, defined as *R* =[*V_out_DC_CO_* − *V_out_DC_air_*/*V_out_DC_air_*] × 100, with and without lock-in amplifier is shown in Figure 19. The response is increased in more than one order of magnitude with the LIA, as it not only amplifies the amplitude of the signal but also reduces noise contribution.

Based on the LIA output voltage, the sensor resistance (*R_s_*) can be determined from the following expression:

(3) $$R_{s}=R_{\text{ref}}\left(\frac{2AV_{in}}{\pi \cdot {\text{Vout}}_{DC\_\text{LIA}}}\cos\theta -1\right)$$

The equivalent resistance of the sensor estimated from (3) is shown in Table 4.

## 5. Conclusions

A novel integrated 1.8 V–0.18 μm CMOS lock-in amplifier with reduced area and power consumption was presented in this paper. It is based on synchronous rectification of the signal in the current domain. The architecture programmability allows for gain adjustment according to the amplitude of the incoming signal, thus providing versatility and flexibility to the circuit for its use in several applications. Moreover, its bandwidth can also be adjusted to further attenuate unwanted signals and noise, improving accuracy in measurements.

Experimental results show that the proposed LIA is able to recover a signal of interest noisy environments with a relative error below 4.1% for a SNR = −42.13 dB. Power consumption is 417 μW and integration area is 0.013 mm^2^, which means a reduction of one order of magnitude in power consumption and two orders of magnitude in the integration area compared to other implementations found in the literature. All these features make it a preferable choice in portable sensing applications.

Finally, the proposed LIA was used for the detection of small concentrations of carbon monoxide with a commercial CO sensor. The response to CO was increased in more than one order of magnitude when compared to a single voltage divider interface circuit. The functionality of the LIA was thus demonstrated.

Future work includes the integration of a phase control, to ensure the in-phase condition for proper operation of the LIA, as well as integration of the LPF output stage, in order to achieve a fully integrated solution.

Although the digitalization and actuation system depends on the final application, in a complete sensor system, the proposed analog lock-in can be the core of a general purpose microcontrolled-based portable low-power electronic interface, so that the μC controls all the measurement process and, if possible, the ADCs in the μC perform the final digitalization, with the measured data transmitted via RF. Alternatively, instead of classical ADCs, voltage-to-frequency converters (VFCs) can be used as a more suitable solution for μC-based applications.

## Figures and Tables

**Table 1.** Gain of the LIA for each digital control word.

***a*_0_**	***a*_1_**	***a*_2_**	***A_id_*(*dB*)**	***A_exp_*(*dB*)**	***BW_exp_*(kHz)**
0	0	0	40.0	42.0	125
0	0	1	38.8	41.0	128
0	1	0	37.5	38.0	122
0	1	1	36.0	36.9	123
1	0	0	34.0	33.0	126
1	0	1	31.5	32.2	126
1	1	0	28.0	27.1	125
1	1	1	22.0	24.7	125

**Table 2.** Signal Recovery Test Results.

**Type of Noise**	**Cases**	***V_out_dc_exp_*(V)**	***C_ext_* = 34 pF *BW_LIA_* = 125 kHz**		***C_ext_* = 254 pF *BW_LIA_* = 13 kHz**	
	
			***V_out_dc_noisy_* (V)**	**Relative Error**	***V_out_dc_noisy_* (V)**	**Relative Error**
Interference	Case 1(a) *ν_s_*=50 μV_pp_ @1 kHz *ν_no_*=6.4 mV_pp_ @2 kHz	0.8964	0.9017	0.6%	0.9010	0.5%

	Case 1(b) *ν_s_*=50 μV_pp_ @1 kHz *ν_no_*=6.4 mV_pp_ @1.025 kHz	0.8964	0.9028	0.7%	0.9021	0.6%

	Case 1(c) *ν_s_*=50 μV_pp_ @1 kHz *ν_no_*=6.4 mV_pp_ @1.005 kHz	0.8964	0.9083	1.3%	0.9074	1.2%

	Case 1(d) *ν_s_*=50 μV_pp_ @1 kHz *ν_no_*=6.4 mV_pp_ @1.002 kHz	0.8964	0.9223	2.9%	0.9217	2.8%

	Case 1(e) *ν_s_*=50 μV_pp_ @1 kHz *ν_no_*=6.4 mV_pp_ @0.998 kHz	0.8964	0.9222	2.9%	0.9217	2.8%

	Case 1(f) *ν_s_*=50 μV_pp_ @1 kHz *ν_no_*=6.4 mV_pp_ @0.995 kHz	0.8964	0.9083	1.3%	0.9075	1.2%

Gaussian White Noise	Case 2 *ν_s_*=17.7 μV_rms_ @1 kHz *ν_no_*=2.3 mV_rms_	0.8964	0.9411	5.0%	0.9332	4.1%

**Table 3.** LIA Electrical Characterization.

**Parameter**	**Proposed LIA**	**Reference [7], 2010**	**Reference [11], 2009**	**Reference [12], 2010**
CMOS Technology (μm)	0.18	0.35	0.18	0.35
Supply Voltage (V)	1.8	±1	1.8	3.3
Gain (dB)	24.7–42	10–110	-	-
BW (kHz)	0.6–125	-	100	13–25
SNR {*ε* <4.1 %} (dB)	−42.13 ^a^	-	-	−1.31
Resolution (μV_pp_)	50	1	-	-
Power Consumption (mW)	0.417	3	2	12.79
Integrated Area (mm^2^)	0.013	5	2	1.5

a with *C_ext_* = 254 pF.

**Table 4.** Gas sensor resistance estimation.

**Sensor Characterization**			**LIA Output**	
CO + N_2_ (ppm)	*t_sens_*(min)	*period*(s)	*Vout_DC_LIA_* (mV)	*Rs_LIA_* (kΩ)
0	3.0	0–180	959.14	19.820
1	28.0	181–1860	962.93	15.557
0	46.0	1861–4620	958.33	20.802
2	29.0	4621–6360	964.40	14.040
0	83.0	6361–11,340	957.77	21.499
3	16.0	11,341–12,300	969.02	9.683
0	35.5	12,301–14,430	955.19	24.890
